# Sudden onset headaches in paediatric emergency departments: diagnosis and management

**DOI:** 10.1186/s13052-023-01526-4

**Published:** 2023-09-14

**Authors:** Léa Lenglart, Cécile Monteil, Eugenia Spreafico, Thomas Moulding, Luigi Titomanlio

**Affiliations:** 1 Paediatric Emergency Department, APHP - Hopital Robert Debré, 48 Boulevard Serurier, Paris, 75019 France; 2 Paediatric Department, IRCCS Fondazione Policlinico S. Matteo, University of Pavia, Pavia, Italy; 3 Paediatric Department, Leeds Teaching Hospitals Trust, Leeds, UK; 4 Paediatric Migraine and Neurovascular Diseases Unit, APHP - Hopital Robert Debré, Paris, France; 5 Paris University, INSERM U1141, DHU Protect, Paris, France

**Keywords:** Paediatric emergency department, Sudden onset headache, Primary headaches, Children, Primary stabbing headaches, Migraine

## Abstract

Headache is one of the most common pain syndromes in the paediatric population. Headaches are classified as primary (migraine, tension-type headaches, trigeminal autonomic cephalalgia and other primary headaches) or secondary (e.g. post-traumatic). Non-febrile, non-traumatic headaches represent 1% of all paediatric emergency departments (PED) visits. Many patients present with an acute, moderate to severe pain, sometimes with a sudden onset. Sudden onset headache can be the main symptom of life-threatening neurological conditions as well as a sign of primary headaches such as thunderclap or stabbing headaches. This review aims to describe the presentation of sudden primary headaches in children, in order to help the physician to provide effective management in the emergency setting.

## Introduction

The Global Burden of Disease Study ranked migraine and tension-type headaches (TTH) as the sixth leading cause of years lived with disability in 2016 [1]. The prevalence of primary headaches is age-related, meaning that school-aged children are less affected than adolescents 2, 3. However, more than one child in two is affected each year by headaches 3.

The International Classification of Headaches Disorders (ICHD) differentiates primary from secondary headaches 4. Primary headaches are disorders in their own right. They are caused by independent pathological mechanisms and not by other disorders. Primary headaches are divided into four categories: migraine, TTH, trigeminal autonomic cephalalgias and other primary headache disorders. The latter category includes ten less common diagnoses: cough headache, exercise headache, headache associated with sexual activity, thunderclap headaches, cold-stimulus headache, external-pressure headache, primary stabbing headache, nummular headache, hypnic headache, new daily persistent headache.

Primary headaches in children are a frequent cause of consultation to primary care physicians and paediatric emergency departments (PED). Indeed, all types of headaches represent between 0.7% and 2.0% of all paediatric consultations to the PED 5–7. A sudden onset headache, a particularly painful headache or an unusual course of an already diagnosed primary headache is usually the reason. When in the PED, identifying secondary headaches due to serious or life-threatening causes is a principal concern for physicians 8. However, a very low percentage of secondary headaches corresponds to serious illnesses 9–12, such as those caused by a raised intracranial pressure including intracranial tumours, meningitis or cerebrovascular disease. Diagnosis often proves challenging and has led to a high use of cranial computed tomography (CT) scans. An increased risk of developing a brain malignancy or a leukaemia in the years following a head CT scan has been identified in the paediatric population 13, 14. Thus, the risk should be balanced in order to avoid the unnecessary use of imaging in the paediatric population.

Primary headaches of sudden onset such as trigeminal autonomic cephalalgia (TACs), thunderclap headache and primary stabbing headache are thought to be under-diagnosed in children. Patients frequently experience a significant delay between the onset of symptoms and the diagnosis, leading to a major impact on their quality of life 15, 16.

The aim of this review is to present an up-to-date review of the different types of sudden onset headaches, and the red flags to search for in the PED.

## Methodology

Electronic database searches of Pubmed and Cochrane Database of Systematic Reviews were performed. The terms, “primary headaches”, “sudden onset headaches”, “migraines”, “tension-type headaches”, “primary stabbing headaches”, “trigeminal autonomic cephalalgia”, “primary thunderclap headaches”, “exercise headaches”, “hypnic headaches”, or “life-threatening headaches” associated to: “children”, “paediatric emergency department” and “diagnosis” were undertaken. All English language publications up to June 2023 were included. Hand searches were also performed, with the reference bibliography from already identified studies, review articles and guidelines screened for additional studies. After removal of duplicate studies and exclusion of non-relevant articles, more than 100 articles were assessed for eligibility. Of these, 64 full-text articles were included in the review.

### Headaches in the PED

A US study reported a 166% increase in visits for headaches in 25 PEDs between 2003 and 2013, compared to an increase in total PED visits of only 57.6% during the same time period 6. In 2013, headaches represented 22.6 per 1,000 PED visits, which correlates with the estimates of other studies (between 0.7% [5] and 2% [7] of all PED visits). The mean age of patients presenting to the PED was between 9 [5, 10, 11, 17, 18] and 12 [6, 7] years. Some studies reported a preponderance of girls 7, 17, while others a preponderance of boys 5, 18.

Regarding the aetiologies of headaches, primary headaches represent between 10% [5] and 75% [19] of all headaches visits at the PED. Among them, migraine and TTH were the most prevalent pathologies, while other primary headaches were rarely reported 17.

Secondary headaches are mostly non-neurological and secondary to upper respiratory tract infections (URTI) such as pharyngitis, sinusitis, otitis media or viral infections such as influenza. Indeed, these infectious non-neurological aetiologies represent between 26.6% [10] and 59.9% [18] of all headache visits depending on the study. Serious or life-threatening neurological illnesses are rare, estimated between 1% [18] and 6% [17] of all PED visits for headaches. Aetiologies sorted by the type of onset are presented in Table 1.

**Table 1:** Type of onset of headaches and etiology of headaches

SUDDEN ONSET HEADACHES	RECURRENT OR CHRONIC PROGRESSIVE HEADACHES
**Primary headaches** - Migraine - Tension-type headaches (TTH) - Primary stabbing headaches (PSH) - Primary Thunderclap headaches	**Primary headaches:** - Chronic migraine - Tension-type headaches - Primary stabbing headaches - Cluster headaches
**Non-neurological secondary headaches** - Upper respiratory tract infection (URTI) - Acute sinusitis - Hypertension - Substance or drug abuse - CO intoxication	**Non-neurological secondary headaches** - Hypertension - Hyperthyroidism - Phaeochromocytoma - Medication-induced headache
**Neurological secondary headaches** - Viral or bacterial meningitis - Subarachnoid or intracranial haemorrhage - Venous sinus thrombosis - Reversible vasoconstriction syndrome - Acute hydrocephalus (including ventriculoperitoneal shunt malfunction)	**Neurological secondary headaches** - Seizures - Hydrocephalus - Chronic subdural haematoma - Unruptured vascular malformation - Brain tumour or abscess - Pseudotumour cerebri

Whilst it is most important for a physician to rule out a diagnosis of secondary headaches due to life-threatening aetiologies, it is important to recognise that, in addition to their low prevalence, these pathologies are usually accompanied by focal neurological abnormalities: papilloedema, blurred vision, ataxia, hemiparesis, cranial nerve paralysis, or other impairment, mostly visual 10, 11, 17, 18. Red flags have been identified in some studies 12, 17 focusing on the characterisation of the headache itself. Occipital headaches, headaches waking patients up from sleep or manifesting soon after waking up, headaches increasing in frequency, duration or severity, and headaches accompanied by vomiting are most associated with serious neurological illnesses. Interestingly, a recent study of sudden non-traumatic headaches in children aged under 6 found a low prevalence of urgent intracranial conditions, downgrading the value of age as a red flag 20. A summary of reported red flags is presented in Table 2.

**Table 2:** Anamnestic and clinical red flags in sudden onset headaches

Anamnestic red flags
- High-risk population: sickle cell anaemia, malignancy, ventricular-peritoneal shunt, neurocutaneous disease, coagulopathy etc. - Age < 5 years - Recent changes in mood or personality - Recent head trauma - Altered general condition, weight loss - Occipital pain - Pain that wakes the child from sleep or worst when waking up, - Pain worsened by coughing or Valsalva maneuver. - Changes in headache characteristics of a child diagnosed with primary headaches - Association with severe vomiting, particularly in the morning - Blurred vision or gait abnormalities - Seizures
**Physical examination red flags**
- Altered conscious state - Meningism - Visual field defects, papilloedema, abnormal ocular movements, pathological pupillary responses - Focal neurological deficit - Cranial nerve palsy - Ataxia, walking abnormalities, impaired coordination - Increased head circumference - Hypertension, bradycardia

In the PED setting, the most frequent neuroimaging performed for headache is the CT scan of the brain; between 8.8% [10] and 36% [6] of children underwent either CT or Magnetic Resonance Imagery (MRI) of the brain as part of their diagnostic work-up for headache. Interestingly, the frequency of neuroimaging has decreased over the last 10 years 6, 7. Perry et al. estimated the decrease to be -3.7% each year between 2007 and 2014, declining from 36 to 14% by 2014 [6]. The most recent studies 6, 12 both report the increasing use of brain MRI in the last years, due to its increased availability in an emergency setting. Rossi et al. reported the lowest rate of neuroimaging (8.8%); of those investigated, only 12.5% had pathological findings, demonstrating that neuroimaging in children with headache is still overused in the PED 10.

Among primary headaches, those with a sudden and severe onset are the most frequently seen in the PED: migraine, TTH, trigeminal autonomic cephalalgias (TACs), primary stabbing headache and primary thunderclap headaches. Exercise or hypnic headaches can also present as sudden onset headaches but they are extremely rare in the paediatric population. Particularly, exercise headaches diagnosis is challenging as frequently misdiagnosed as migraine, but they differ in the fact that migraine are aggravated and not provoked by exertion 21.

### Sudden onset primary headaches

#### Migraine with and without aura and tension-type headaches

Migraine in children can arise with or without an aura, similarly to adults. However, they differ from those experienced by adults; therefore, the diagnostic criteria are different 4. The ICHD-3b defines migraine without aura in the paediatric population as a recurrent headache disorder manifesting as attacks lasting between 2 and 72 h. Migrainous headaches are typically unilateral, pulsatile, of moderate to severe intensity, aggravated by physical activity, and associated with nausea, photophobia and phonophobia. Auras are defined as recurrent attacks of unilateral and fully-reversible visual, sensory or other central nervous system symptoms that usually develop gradually, last for several minutes and usually precede the headache and associated symptoms. Migraine with aura may have either a prodromal or post-dromal phase, characterised by hyperactivity or hypoactivity, depression, and fatigue. The features of migraine often differ to those seen in adults: they are commonly bilateral (typically unilateral in adults), frontal (ocular or temporal in adults) 22, non-pulsatile (especially in young children) 23 and shorter (sometimes less than 30 min, especially in young children) 21, 24. Moreover, migraines with aura are more common in adolescents than in younger children; This may be due to the difficulties children often face when describing their symptoms 21. Making a positive diagnosis of migraine in the PED and referring the patient to a headache outpatient clinic has been shown to decrease further PED visits for migraine crises 11.

Interestingly, in their personal medical history, children presenting with migraines are more likely to have suffered from infantile colic 25, regurgitation 26, abdominal migraine, cyclic vomiting syndrome and other disorders of the gut-brain interaction 27, 28. Indeed, infantile colic, cyclic vomiting syndrome and abdominal migraine have now been recognized as predictors of migraine 29. Moreover, as migraine has a genetic heritability component, most children who suffer from migraine have a family history of migraine 30.

TTHs, as defined by the ICHD-3b, are episodes of headache, typically bilateral, pressing or tightening, of mild to moderate intensity, lasting anywhere from minutes to days. The pain does not worsen with routine physical activity and is not associated with nausea, although photophobia or phonophobia may be present. Thus, children can continue to play despite their headache. TTHs are defined according to their frequency and can thus be qualified as infrequent (< 1 day per month on average), frequent (1–14 days per month on average for > 3 months) or chronic (≥ 15 days per month on average for > 3 months).

The precise diagnostic criteria of the ICHD-3b for migraine and TTH are presented in Table 3.

**Table 3:** Definition of migraine, tension type-headaches, primary stabbing headaches and thunderclap headaches, according to the International Classification of Headaches Disorders – 3b

MIGRAINE WITHOUT AURA	MIGRAINE WITH AURA
A. At least five attacks fulfilling criteria B-D B. Headache attacks lasting 2–72 h (untreated or unsuccessfully treated) C. Headache has at least two of the following four characteristics: - unilateral location - pulsating quality - moderate or severe pain intensity - aggravated by or causing avoidance of routine physical activity (e.g. walking or climbing stairs) D. During headache, at least one of the following: - nausea and/or vomiting - photophobia and phonophobia E. Not better accounted for by another ICHD-3 diagnosis.	A. At least two attacks fulfilling criteria B and C B. One or more of the following fully reversible aura symptoms: - Visual - Sensory - speech and/or language - motor - brainstem - retinal C. At least three of the following six characteristics: - at least one aura symptom spreads gradually over ≥ 5 min - two or more aura symptoms occur in succession - each individual aura symptom lasts 5–60 min - at least one aura symptom is unilateral - at least one aura symptom is positive - the aura is accompanied, or followed within 60 min, by headache D. Not better accounted for by another ICHD-3 diagnosis.
**TENSION-TYPE HEADACHES (INFREQUENT)**	
A. At least 10 episodes of headache occurring on < 1 day/month on average (< 12 days/year) and fulfilling criteria B-D B. Lasting from 30 min to 7 days C. At least two of the following four characteristics: - bilateral location - pressing or tightening (non-pulsating) quality - mild or moderate intensity - not aggravated by routine physical activity such as walking or climbing stairs D. Both of the following: - no nausea or vomiting - no more than one of photophobia or phonophobia E. Not better accounted for by another ICHD-3 diagnosis1.	
**CLUSTER HEADACHES and other Trigeminal Autonomic Cephalalgias (TACs)**	
A. At least five attacks fulfilling criteria B-D B. B. Severe or very severe unilateral orbital, supraorbital and/or temporal pain lasting 15–180 min (when untreated) C. C. Either or both of the following: a. at least one of the following symptoms or signs, ipsilateral to the headache: - conjunctival injection and/or lacrimation - nasal congestion and/or rhinorrhoea - eyelid oedema - forehead and facial sweating - miosis and/or ptosis b. a sense of restlessness or agitation D. Occurring with a frequency between one every other day and 8 per day E. Not better accounted for by another ICHD-3 diagnosis. **Paroxysmal Hemicrania (compared to cluster headaches)**: A differs: at least 20 attacks B differs: lasting 2–30 min and occurring several or many times a day C is the same D differs: Occurring with a frequency of > 5 per day E: Prevented by therapeutic doses of indomethacin F: not better accounted by another ICHD-3 diagnosis **Short-lasting unilateral neuralgiform headache attacks - SUNCT/SUNA (compared to cluster headaches)**: A differs: at least 20 attacks B differs: Moderate or severe pain, lasting for 1–600 s C differs: forehead and facial flushing and sensation of fullness in the ear in addition to the above symptoms D differs: Occurring with a frequency of at least one per day E is the same	
**PRIMARY STABBING HEADACHES**	**THUNDERCLAP HEADACHES**
A. Head pain occurring spontaneously as a single stab or series of stabs and fulfilling criteria B and C B. Each stab lasts for up to a few seconds C. Stabs recur with irregular frequency, from one to many per day D. No cranial autonomic symptoms E. Not better accounted for by another ICHD-3 diagnosis.	A. Severe head pain fulfilling criteria B and C B. Abrupt onset, reaching maximum intensity in < 1 min C. Lasting for ≥ 5 min D. Not better accounted for by another ICHD-3 diagnosis

### Trigeminal autonomic cephalalgias

TACs are a group of three primary headache disorders including cluster headaches (CH), paroxysmal hemicrania (PH) and short-lasting unilateral neuralgiform headache with conjunctival injection and tearing/cranial autonomic features (SUNCT/SUNA). They are defined by the ICHD-3b 4 as attacks of severe and strictly unilateral pain, which is orbital, supraorbital, temporal or any combination thereof. The pain is associated with ipsilateral conjunctival injection, lacrimation, nasal congestion, rhinorrhoea, forehead and facial sweating, miosis, ptosis and/or eyelid oedema, and/or with restlessness or agitation. They differ in duration, frequency and rhythmicity of the attacks, which can be particularly challenging to discriminate in the paediatric population 31 due to children’s difficulty to precisely describe their pain 32. One key to the diagnosis of PH is the efficacy of indomethacin in preventing the recurrence of attacks. The diagnostic features of TACs can be found in Table 3.

The yearly prevalence of CH in a tertiary outpatient paediatric neurology clinic has been estimated at around 0.03%, which is very low 33. TACs are primarily diagnosed in school-aged children (between 6 and 13), but patients have often been suffering from attacks for 12–24 months prior to the diagnosis. The mean age at diagnosis is between 9 and 13 years, with the onset of symptoms typically reported between 8 and 10 years 32, 34–38; however, some very early cases of TACs have been described in the literature 39–41, the youngest being diagnosed at the age of 2 after more than 6 months of PH attacks 39. A male predominance has been described as well as a family history of TACs or other primary headache disorders 32, 34, but this is not demonstrated in all reviews. The pain associated with TACS mainly corresponds to the ICHD-3b criteria, although some patients describe bilateral or alternating-side pain 32, 37. Moreover, migrainous features, such as nausea, vomiting, phonophobia and –photophobia, may be present in some patients, reinforcing the connection between these entities 32. Potential triggers of PH include heat waves, stress, tobacco smoke and exercise.

The successful treatment of PH with indomethacin is a defining feature and treatment is usually necessary for 1 to 2 years before it can be definitively stopped 32, 42. However, a case report describes an 11 year-old Japanese patient treated with indomethacin for PH after only 5 days of experiencing attacks, who was able to stop treatment after just 14 days 43. Gastrointestinal and behavioural side effects of indomethacin are common 42 and can lead to discontinuation of treatment; therefore, this case indicates the importance of improving awareness regarding PH, since early treatment could prevent long-course therapy. Verapamil is the prophylactic treatment of choice for CH although many other medications can be used: corticosteroids (short-term), lithium, topiramate, gabapentin, indomethacin, and sodium valproate 34, 44. The most common and effective treatment for acute CH attacks are oxygen inhalation via a non-rebreathe facial mask at a flow rate of 7 L/minute for more than 15 min, or sumatriptan nasal spray. 34, 44.

### Primary stabbing headache

PSH is defined by the ICHD-3b as transient (up to a few seconds) and localized stabs of pain in the head that occur spontaneously, in the absence of any organic disease of underlying structures or of the cranial nerves. The precise diagnostic criteria of the ICHD-3b for PSH are presented in Table 3. PSH in the paediatric population is considered to be a very rare pathology, and its prevalence among sufferers of chronic headaches is estimated between 1.5% [45] and 3.5% [46] in cohort studies of up to 83 patients 47.

Mean age at diagnosis is around 9 years old 46–49 and patients usually suffer from stabs for a mean of 6 [48] to 11 months before being diagnosed. Comparatively to adults, a female gender predominance has not been clearly established in the paediatric population 50. However, as in most chronic headache disorders, a family history of chronic headaches can be found in up to 58% of the patients in different cohorts 46–48, 51.

In the paediatric population stabs can last for more than a few minutes in up to one third of patients 46, 47, 51.This suggests the ICHD-3b criteria of PSH may need to be modified for children and adolescents 15. Stabs can occur over any region of the scalp. Soriani et al. found that attacks occurred more than once a week in 52% of patients, once a week in 21% and once a month for the remainder 47. PSH is usually treated with either indomethacin or anti-epileptic therapies; however, no proof of efficacy for either treatment has been made to date 52. Furthermore, patients who receive no therapeutic treatment usually see a resolution of symptoms within a few months or years 46, 48.

### Primary thunderclap headache

Primary thunderclap headache is defined by the ICHD-3b as a high-intensity headache of abrupt onset, mimicking that of a ruptured cerebral aneurysm, in the absence of any intracranial pathology. (See Table 3 for precise criteria of ICHD-3b definition of thunderclap headaches). In the paediatric population, only one retrospective study reports a cohort of patients with primary thunderclap headaches, presenting to the PED of a tertiary-care hospital over a period of two years 53. Thunderclap headaches accounted for 0.8% of all headaches, with a 10/10 pain score. Among them, 42% were diagnosed with primary thunderclap headaches, which is much higher than the prevalence of primary thunderclap headaches in the adult population of thunderclap headaches 54. The higher proportion of primary thunderclap headaches is most likely due to the low incidence of neuro-vascular diseases, such as subarachnoid haemorrhage, cervical artery dissection, reversible cerebral vasoconstriction syndrome and cerebral venous sinus thrombosis 53, 55.

### Life-threatening secondary headache of sudden onset

As previously discussed, headaches in the PED are mainly due to non-life-threatening conditions such as URTI or primary headaches. However, it is still essential for clinicians to be cognizant of potentially life-threatening acute secondary headaches. Headache in these situations is secondary to the rise of intracranial pressure, caused either by an excess of cerebrospinal fluid, blood or oedema, or the presence of a mass, cyst or aneurysm. Such pathologies are mainly diagnosed in patients whose history and physical examination reveal “red flags” (see Table 2). A thunderclap headache is defined as a severe and acute headache that typically reaches its peak within a minute and lasts for about 5 minutes 55. Diagnoses to be considered in this rapidly evolving situation are: spontaneous intracranial haemorrhage; cervical arterial dissection; venous sinus thrombosis; reversible cerebral vasoconstriction syndrome, and acute hydrocephalus.

A recent review on the causes and risk factors of spontaneous intracranial haemorrhage in children, such as subarachnoid haemorrhage, found vascular aetiologies to be responsible in 43% of cases, with arteriovenous malformations being the most frequent. Haematological and systemic causes, cardiac causes and intra-cranial infections are rarer aetiologies of spontaneous intracranial haemorrhage 56. These patients present with abnormal physical examination in most cases 11, 17, 18, 55. Regarding cervical artery dissection, the location of the vascular wall laceration determines the symptoms. Thus, the classic dissection of carotid or vertebral vessels presents with acute onset headache associated with neck pain and/or tenderness and ipsilateral supraorbital, auricular, or mandibular pain. Focal neurological abnormalities on physical examination are frequently present as well 57, 58. When suspected, CT or MRI angiography of the head and neck are the preferred imaging methods.

The clinical manifestations of cerebral sinus venous thrombosis include headache, seizures, lethargy, nausea and vomiting, and focal neurological abnormalities. They are non-specific and may be subtle, often overlapping with the clinical presentation of its risk factors (head and neck infections, dehydration, anaemia, autoimmune disorders, malignancy etc.) 59. Imaging of cerebral sinus venous thrombosis require a head CT or MRI with and without contrast agent. The superior sagittal and transverse sinuses are most frequently involved 60.

Reversible cerebral vasoconstriction syndrome (RCVS) is an acute and transient malfunction of the intracranial vascular tone, which causes diffuse vasoconstriction of the cerebral arterial vasculature and is associated with recurrent severe headaches. In most cases of RCVS in both children and adults, triggering factors can be identified such as trauma, exercise, water to the face, hypertension, and medication or substance use. Repeat imaging, including vascular imaging or transcranial Dopplers, may be required before one can exclude the diagnosis, particularly if the imaging was performed within a few days of the trigger or initial headache. Given the reversible nature, most patients experience a full recovery of symptoms 61, 62.

Moreover, acute hydrocephalus, caused by the compression of a tumour or a cyst, an infection or a ventriculoperitoneal shunt malfunction can cause a sudden onset headache and will be associated with anamnestic or clinical red flags. Besides, although headaches are most often associated to the diagnosis of meningitis, they are not uncommon in encephalitis cases, where fever may be modest 63.

Finally, when evaluating a child presenting with a sudden onset headache, rare but life-threatening aetiologies of headaches should be considered and ruled out before making the diagnosis of primary headache of sudden onset.

### Diagnosis management of sudden onset headaches at the PED

Physical examination and history of the headache are of crucial importance in the evaluation of sudden onset headaches at the PED.

#### History

A precise description of the headache is necessary, including the onset, duration, localisation, quality, pain severity, association with aura or any other neurological symptoms and triggers, as well as investigating any red flags. Although it is not the subject of this review, chronic progressive headaches are particularly concerning, as well as recent trauma or an association with fever, all of which need to be ruled out.

Personal medical history (primary headaches, neurocutaneous disorders, congenital heart disease, sickle cell disease, coagulopathies, peritoneal ventricular shunts) and family history also need to be elicited 8, 55. A HEADSS (home, education, alcohol, drugs, smoking and sex) screening should be part of the evaluation of every adolescent presenting with headaches as well as enquiring about psychosocial, school and domestic stressors 64.

#### Physical examination

A complete physical examination and a precise neurological examination should be performed, as well as an assessment of pain severity. Particular attention should be paid to the level of consciousness, visual disturbances and pupillary reactivity, ataxia and coordination disorders, focal neurological deficits or altered sensitivity, meningitic signs, and head circumference for babies. A vital signs assessment, including blood pressure, should also be performed.

#### Neuroimaging and other investigations

CT or MRI are the most frequent investigations performed in the PED in cases of sudden onset headaches, as they allow the physician to eliminate most of the life-threatening diagnoses of secondary headaches. However, they should only be reserved for children with anamnestic or clinical red flags 8, 12, 55. CT scan with or without a contrast agent is the imaging modality of choice in an emergency setting, even though an MRI with the right sequences (MRA, T2-weighted gradient-echo, diffusion-weighted sequences and post-gadolinium enhanced sequences) would be the most accurate imaging in this context.

In addition to the use of neuroimaging, electro-encephalogram can be useful in specific cases such atypical epileptic seizure presenting with per-ictal headaches or atypical auras of migraines 8. Laboratory blood tests are rarely useful in the evaluation of sudden onset headaches. In cases of a suspected intracranial infection, subarachnoid haemorrhage or idiopathic intracranial hypertension, a lumbar puncture is recommended after neuroimaging 55.

### Strength and limits of the study

We performed a systematic and thorough search of all available data on sudden onset primary headaches in the PED from different sources in order to limit the selection bias inherent to narrative reviews. However, articles that were not published in English could not be included.

## Conclusion

Most sudden onset headaches in the PED setting are non-life-threatening headaches, such as primary headaches or secondary to URTI.

Among primary headaches, although migraine is well known by PED physicians, other rarer syndromes deserve attention, notably stabbing headaches and thunderclap headaches. These can be differentiated by taking a typical headache history using ICHD criteria. On the other hand, life-threatening conditions must be ruled out and can be diagnosed in the PED by following anamnestic and physical criteria (headache red flags).

## Data Availability

Doesn’t apply.

## Abbreviations

PED: Paediatric Emergency Department
TTH: Tension Type Headaches
ICHD: International Classification of Headaches Disorders
CT: Computed Tomography
TACs: Trigeminal Autonomic Cephalalgia
URTI: Upper Respiratory Tract Infection
MRI: Magnetic Resonance Imagery
CH: Cluster Headaches
PH: Paroxysmal Hemicrania
SUNCT/SUNA: Short-lasting Unilateral Neuralgiform headache with Conjunctival injection and Tearing/cranial Autonomic features
RCVS: Reversible Cerebral Vasoconstriction Syndrome
